# Interleukin gene polymorphisms in Chinese Han population with breast cancer, a case-control study

**DOI:** 10.18632/oncotarget.23157

**Published:** 2017-12-11

**Authors:** Xiaoxiao Zuo, Miao Li, Ya Yang, Tiansong Liang, Hongyao Yang, Xinhan Zhao, Daoke Yang

**Affiliations:** ^1^ Department of Radiation Oncology, First Affiliated Hospital of Zhengzhou University, Zhengzhou, Henan Province 450000, People's Republic of China; ^2^ Department of Internal Medicine Oncology, The Fifth People's Hospital of Qinghai Province, Xining, Qinghai 810007, China

**Keywords:** cytokines, IL1, breast cancer, case-control, polymorphisms

## Abstract

Cytokines are known as important regulators of the cancer involved in inflammatory and immunological responses. This fact and plethora of gene polymorphism data prompted us to investigate IL1 gene polymorphisms in breast cancer (BC) patients. Totally, 530 patients with BC and 628 healthy control women were studied. The genetic polymorphisms for IL1 were analyzed by Massarray Sequencing method. Three single nucleotide polymorphisms (SNPs) identified in IL1B, IL1R1 gene are thought to influence breast cancer risk. The results of the association between IL-1B, IL1R1 polymorphisms and breast cancer risk have significant. We found that the variant TT genotype of rs10490571 was associated with a significantly increased breast cancer risk (TT vs. CC: OR = 2.82, 95% CI = 1.12–7.08, *P* = 0.047 for the codominant model). For rs16944 (AG vs. GG: OR = 0.60, 95% CI = 0.41–0.90, *P* = 0.034 for the codominant model) and rs1143623 (CG vs. CC: OR = 0.65, 95% CI = 0.45–0.94, *P* = 0.023 for the codominant model) have significant associations were found in genetic models. In conclusion, the present analysis suggests a correlation of polymorphic markers within the IL-1 gene locus with the risk in developing breast cancer. Taken together with our finding that IL1B, IL1R1 gene three SNP are also associated with the risk for the disease, we suggest that inflammation via innate and adaptive immunity contributes to multifactorial hereditary predisposition to pathogenesis of the breast cancer.

## INTRODUCTION

Breast cancer (BC) is one of the most common causes of cancer death in women and one of the important factors contributing to the whole world health burden [1]. Currently, routine BC clinical management depended on few well-defined biologicalmarkers and clinicopathological variables. Mutations in BRCA1, BRCA2 gene is inherited in an autosomal dominant manner and possess highly risky [2, 3] however, account for only a small percentage of BC cases [4]. It is very likely that a number of low penetrance genes conduce to BC susceptibility, thereby accounting for a higher proportion of the disease burden [5, 6].

The role of cytokines in cancer immunization and carcinogenesis has been recognized [7]. Many studies of cytokine polymorphisms associated with susceptibility to [8, 9], liver cancer [10, 11], lung cancer [12], prostate cancer [13] and ovarian cancer [14] are mixed.

Interleukin (IL) −1 is an endogenous cytokine family involved in inflammation and immune response, including IL-1 alpha (IL-1A), IL-1 beta (IL-1B) and IL-1 receptor antagonist (IL-1Ra) encoded by IL-1RN [15]. IL-1 is one of the major proinflammatory cytokines that increases in cancer patients [16]. In many tumor types, IL-1 is thought to be up-regulated and is expressed as a factor in tumor progression by metastasis and expression of angiogenic genes and growth factors.

Many studies have reported that high IL-1 concentrations in the tumor microenvironment are associated with more toxic tumor phenotypes. For example, in solid tumors, IL-1 has been shown to be upregulated, including melanoma, colon cancer, lung cancer, head and neck cancer, and patients with high IL-1 producing tumours have generally bad prognoses [17, 18]. IL-1 has been recently suggested to play a role in the development of breast cancer. Multifunctional cytokines are closely related to the development of inflammatory and immunological responses which play a key role in the pathogenesis of autoimmune and malignant diseases [7, 19], making them an important candidate genes in BC. Considering the diverse roles of cytokines in cell growth, proliferation, differentiation and migration in inflammation and cancers, we proposed that polymorphisms in the cytokine genes could affect the risk of BC. Therefore the present study, we attempt to investigate the selected SNPs possible correlations the BC risk in Han Chinese population.

## RESULTS

This hospital-based case–control study consisted of 530 patients and 628 healthy age-matched controls. Of the total 1158 breast cancer cases, the age of the case subjects was 50.69 ± 11.74, the control was 51.04 ± 9.64. Based on menopause status,

40.8% cases were premenopausal, 59.2% cases were postmenopausal. 43.8% cases were in stages I and 56.2% were II, III and IV. Of these cases, 41.5%, 58.5% belonged to ER^−^, ER^+^, respectively. PR^−^, PR^+^ is 52.5%, 47.5% respectively. Information on status of HER2 expression, 39.2% cases were negative, and 60.8% were positive (Table 1).

**Table 1:** Basic characteristic of patients with breast cancer and the control individuals

Characteristics	Cases (*N* = 530)	Controls (*N* = 628)	*p*-value
Age			0.211
Mean ± SD	50.69 ± 11.74	51.04 ± 9.64	
Menopause			0.909
Premenopausal	216 (40.8%)	258 (41.1%)	
Postmenopausal	314 (59.2%)	370 (58.9%)	
Clinical Stages			
I	232 (43.8%)		
II/III/IV	298 (56.2%)		
Estrogen Receptor			
Negative	220 (41.5%)		
Positive	310 (58.5%)		
Progestrone Receptor			
Negative	278 (52.5%)		
Positive	252 (47.5%)		
HER2			
Negative	208 (39.2%)		
Positive	322 (60.8%)		

The allele and genotype frequencies of IL-1 in the healthy and case groups are shown in Table 2. The control group genotype data for all SNPs were analyzed for fitness in Hardy Weinberg equilibrium with no significant deviation observed in any case. From this table it can be seen that, while no significant allel associations were demonstrated for any SNP. From Table 3, the age-adjusted ORs estimated by a logistic model are shown three SNPs: IL1R1 (rs10490571), IL1B (rs16944, rs1143623) had significant in BC risk. For rs10490571, the estimates relative to the TT genotype were 2.63 (95% CI, 1.05–6.55) for the CC/CT genotype. The rs16944 (A/G) polymorphism was positively associated with the risk of BC in codominant (OR = 0.60, 95% CI = 0.41–0.90; *P* = 0.034), Dominant (OR = 0.67, 95% CI = 0.46–0.97; *P* = 0.035). There was significant association between the rs1143623 C/G allele and BC patients compared to the healthy controls in codominant model (OR = 0.65, 95% CI = 0.45–0.94; *P* = 0.023).

**Table 2:** Basic information of candidate SNPs in this study

SNP	Gene	Chr	Allels(A/B)	p-HWE	MAF		OR	95% CI	*P*
					Case	Control			
rs11674595	IL1R2	2q11.2	C/T	0.408	0.289	0.243	1.16	0.88–1.53	0.217
rs4851527	IL1R2	2q11.2	A/G	0.697	0.382	0.296	0.89	0.70–1.15	0.320
rs719250	IL1R2	2q11.2	T/C	0.103	0.365	0.269	0.89	0.69–1.15	0.293
rs3218896	IL1R2	2q11.2	C/T	0.478	0.997	0.136	1.00	0.71–1.40	0.136
rs3218977	IL1R2	2q11.2	G/A	0.357	0.532	0.226	0.92	0.70–1.20	0.242
rs2072472	IL1R2	2q11.2	G/A	0.129	0.224	0.243	1.19	0.90–1.56	0.213
rs10490571	IL1R1	2q12.1	T/C	0.549	0.026	0.219	1.40	1.04–1.87	0.167
rs956730	IL1R1	2q12.1	A/G	0.352	0.729	0.230	0.95	0.73–1.25	0.239
rs3917225	IL1R1	2q12.1	T/C	0.716	0.217	0.400	1.16	0.92–1.47	0.365
rs3917318	IL1R1	2q12.1	G/A	0.910	0.054	0.442	0.80	0.63–1.00	0.498
rs3783550	IL1A	2q13	T/G	0.532	0.612	0.357	1.06	0.84–1.36	0.342
rs3783546	IL1A	2q13	C/G	0.532	0.612	0.357	1.06	0.84–1.36	0.342
rs2856838	IL1A	2q13	A/G	0.875	0.106	0.275	1.24	0.95–1.62	0.234
rs1609682	IL1A	2q13	T/G	0.532	0.593	0.357	1.07	0.84–1.36	0.342
rs3783521	IL1A	2q13	G/A	0.532	0.612	0.357	1.06	0.84–1.36	0.342
rs2853550	IL1B	2q13	A/G	0.318	0.996	0.092	1.00	0.67–1.49	0.092
rs1143643	IL1B	2q13	C/T	0.910	0.427	0.462	0.91	0.72–1.15	0.486
rs3136558	IL1B	2q13	G/A	0.480	0.593	0.381	0.94	0.74–1.19	0.396
rs1143630	IL1B	2q13	T/G	0.176	0.581	0.166	0.92	0.67–1.25	0.178
rs1143627	IL1B	2q13	G/A	0.142	0.488	0.519	1.09	0.86–1.37	0.498
rs16944	IL1B	2q13	A/G	0.114	0.405	0.475	0.91	0.72–1.14	0.500
rs1143623	IL1B	2q13	G/C	0.132	0.746	0.409	0.96	0.76–1.22	0.419

Abbreviations: SNP: single nucleotide polymorphism, Alleles A/B: Minor/major alleles; MAF, minor allele frequency; OR: odds ratio, CI: confidence interval, HWE: Hardy–Weinberg equilibrium.

*p* ≤ 0.05 indicates statistical significance.

**Table 3:** Logistic regression analysis of the association between the IL1 SNPs and breast cancer risk

SNP ID	Model	Genotype	control	case	OR (95% CI)	*P*-value	AIC	BIC
rs10490571	Codominant	C/C	432 (68.8%)	328 (61.9%)	1			
		C/T	182 (29%)	172 (32.5%)	1.25 (0.87–1.79)	0.047	800.2	817.7
		T/T	14 (2.2%)	30 (5.7%)	2.82 (1.12–7.08)			
	Dominant	C/C	432 (68.8%)	328 (61.9%)	1			
		C/T-T/T	196 (31.2%)	202 (38.1%)	1.36 (0.96–1.92)	0.079	801.3	814.3
	Recessive	C/C-C/T	614 (97.8%)	500 (94.3%)	1			
		T/T	14 (2.2%)	30 (5.7%)	2.63 (1.05–6.55)	0.031	799.7	812.8
	Log-additive	—	—	—	1.40 (1.04–1.87)	0.026	799.4	812.5
rs1143627	Codominant	G/G	144 (22.9%)	136 (25.7%)	1			
		A/G	342 (54.5%)	238 (44.9%)	0.74 (0.49–1.11)	0.054	800.5	818
		A/A	142 (22.6%)	156 (29.4%)	1.18 (0.74–1.87)			
	Dominant	G/G	144 (22.9%)	136 (25.7%)	1			
		A/G-A/A	484 (77.1%)	394 (74.3%)	0.86 (0.59–1.27)	0.45	803.8	816.9
	Recessive	G/G-A/G	486 (77.4%)	374 (70.6%)	1			
		A/A	142 (22.6%)	156 (29.4%)	1.44 (0.99–2.10)	0.056	800.7	813.8
	Log-additive	—	—	—	1.09 (0.86–1.38)	0.47	803.8	816.9
rs16944	Codominant	G/G	142 (22.6%)	160 (30.2%)	1			
		A/G	344 (54.8%)	236 (44.5%)	0.60 (0.41–0.90)	0.034	799.6	817
		A/A	142 (22.6%)	134 (25.3%)	0.83 (0.52–1.32)			
	Dominant	G/G	142 (22.6%)	160 (30.2%)	1			
		A/G-A/A	243 (77.4%)	370 (69.8%)	0.67 (0.46–0.97)	0.035	799.9	813
	Recessive	G/G-A/G	243 (77.4%)	396 (74.7%)	1			
		A/A	142 (22.6%)	134 (25.3%)	1.15 (0.79–1.69)	0.46	803.8	816.9
	Log-additive	—	—	—	0.90 (0.71–1.14)	0.39	803.6	816.7
rs1143623	Codominant	C/C	198 (31.6%)	202 (38.3%)	1			
		C/G	332 (53%)	220 (41.7%)	0.65 (0.45–0.94)	0.023	796.1	813.5
		G/G	96 (15.3%)	106 (20.1%)	1.08 (0.67–1.74)			
	Dominant	C/C	198 (31.6%)	202 (38.3%)	1			
		C/G-G/G	428 (68.4%)	326 (61.7%)	0.74 (0.53–1.05)	0.093	798.8	811.9
	Recessive	C/C-C/G	530 (84.7%)	422 (79.9%)	1			
		G/G	96 (15.3%)	106 (20.1%)	1.38 (0.90–2.13)	0.14	799.4	812.5
	Log-additive	—	—	—	0.96 (0.76–1.21)	0.73	801.5	814.6

Abbreviations: OR: odds ratio, CI: confidence interval AIC: Akaike's Information criterion; BIC: Bayesian Information criterion.

^*^p value < 0.05 indicates statistical significance.

From the stratification analyses, it was found that in Table 4 BC risk associated with rs10490571 T/C variant was more evident in postmenopausal women (OR = 1.46 95% CI = 1.04–2.04, *P* = 0.029). ER^+^ women three SNPs (rs10490571, OR = 1.48 95% CI = 1.06–2.07, *P* = 0.023; rs1143627, OR = 1.38 95% CI = 1.05–1.81, *P* = 0.023; rs16944, OR = 0.73, 95% CI = 0.56–0.96, *P* = 0.026) were associated with BC risk. It is evident that statistically significant association exists between rs10490571, rs3917318, rs2856838, rs1143627, rs16944 polymorphism and BC risk in PR+ women. With regard to HER2 status, the difference was significant, the OR among HER2+ women was 1.59 (1.15–2.22) for rs10490571, 0.72 (0.55–0.94) for rs3917318 were associated with BC risk, whereas that for HER2^−^ women there was no significant SNPs.

**Table 4:** Logistic regression analysis of the association between the IL1 SNPs and breast cancer risk by stratification analysis

SNP ID	Premenopausal			Postmenopausal			ER+			ER-			PR+			PR-			HER2+			HER2-		
	OR	95%CI	*P*	OR	95%CI	*P*	OR	95%CI	*P*	OR	95%CI	*P*	OR	95%CI	*P*	OR	95%CI	*P*	OR	95%CI	*P*	OR	95%CI	*P*
rs11674595	1.29	0.90–1.85	0.158	1.07	0.78–1.49	0.670	1.17	0.85–1.62	0.334	1.14	0.8–1.65	0.465	1.15	0.82–1.63	0.422	1.17	0.84–1.63	0.361	1.19	0.87–1.64	0.275	1.11	0.77–1.61	0.577
rs4851527	0.76	0.54–1.08	0.122	0.99	0.74–1.33	0.961	0.92	0.69–1.24	0.601	0.85	0.61–1.19	0.353	0.93	0.68–1.28	0.676	0.86	0.63–1.17	0.332	0.89	0.66–1.19	0.430	0.90	0.64–1.27	0.554
rs719250	0.95	0.67–1.34	0.767	0.85	0.62–1.15	0.284	0.74	0.54–1.01	0.056	1.13	0.81–1.57	0.483	0.80	0.57–1.11	0.180	0.98	0.71–1.33	0.873	0.94	0.7–1.27	0.706	0.80	0.56–1.15	0.232
rs3218896	1.11	0.71–1.72	0.651	0.93	0.62–1.39	0.721	0.84	0.55–1.27	0.399	1.25	0.82–1.9	0.310	0.86	0.55–1.34	0.506	1.13	0.76–1.69	0.541	1.12	0.76–1.64	0.577	0.83	0.51–1.35	0.450
rs3218977	0.85	0.58–1.23	0.384	0.97	0.7–1.33	0.829	1.07	0.78–1.47	0.669	0.72	0.49–1.06	0.090	1.04	0.74–1.47	0.804	0.81	0.57–1.14	0.223	0.84	0.61–1.16	0.286	1.04	0.73–1.5	0.817
rs2072472	1.26	0.88–1.81	0.210	1.14	0.82–1.57	0.437	1.14	0.82–1.57	0.442	1.26	0.88–1.8	0.208	1.15	0.81–1.63	0.424	1.22	0.87–1.7	0.246	1.28	0.94–1.75	0.123	1.05	0.72–1.53	0.813
rs10490571	1.31	0.89–1.94	0.173	1.46	1.04–2.04	0.029	1.48	1.06–2.07	0.023	1.28	0.87–1.89	0.212	1.73	1.22–2.46	0.002	1.12	0.77–1.62	0.550	1.59	1.15–2.22	0.006	1.11	0.74–1.68	0.607
rs12712127	1.24	0.87–1.78	0.228	1.39	1.02–1.89	0.038	1.34	0.98–1.84	0.062	1.30	0.92–1.85	0.140	1.57	1.14–2.18	0.006	1.12	0.81–1.57	0.490	1.52	1.12–2.06	0.007	1.05	0.73–1.53	0.780
rs956730	0.86	0.59–1.25	0.437	1.02	0.74–1.4	0.914	0.90	0.65–1.24	0.506	1.04	0.73–1.48	0.844	0.87	0.61–1.24	0.435	1.03	0.74–1.43	0.852	0.89	0.64–1.22	0.458	1.06	0.74–1.53	0.745
rs3917225	1.02	0.74–1.41	0.880	1.26	0.96–1.67	0.097	1.13	0.86–1.5	0.390	1.21	0.88–1.65	0.242	1.15	0.85–1.55	0.372	1.18	0.88–1.57	0.273	1.24	0.94–1.64	0.122	1.05	0.76–1.45	0.788
rs3917318	0.75	0.55–1.02	0.066	0.83	0.63–1.09	0.181	0.77	0.58–1.01	0.057	0.84	0.62–1.14	0.263	0.74	0.55–1	0.048	0.85	0.64–1.12	0.249	0.72	0.55–0.94	0.016	0.93	0.68–1.28	0.659
rs3783550	0.98	0.71–1.36	0.906	1.13	0.85–1.49	0.412	1.06	0.79–1.41	0.706	1.08	0.78–1.48	0.654	1.14	0.84–1.55	0.389	1.00	0.74–1.34	0.985	1.04	0.78–1.38	0.792	1.11	0.8–1.53	0.546
rs3783546	0.98	0.71–1.36	0.906	1.13	0.85–1.49	0.412	1.06	0.79–1.41	0.706	1.08	0.78–1.48	0.654	1.14	0.84–1.55	0.389	1.00	0.74–1.34	0.985	1.05	0.79–1.4	0.720	1.08	0.78–1.5	0.632
rs2856838	1.17	0.82–1.67	0.377	1.29	0.95–1.76	0.099	1.26	0.92–1.71	0.148	1.23	0.87–1.74	0.251	1.39	1–1.92	0.050	1.12	0.81–1.56	0.489	1.25	0.92–1.7	0.153	1.24	0.87–1.76	0.245
rs1609682	0.97	0.7–1.35	0.875	1.14	0.86–1.51	0.374	1.07	0.8–1.42	0.655	1.07	0.78–1.48	0.680	1.16	0.85–1.57	0.346	0.99	0.74–1.34	0.959	1.06	0.8–1.41	0.692	1.08	0.78–1.5	0.632
rs3783521	0.98	0.71–1.36	0.906	1.13	0.85–1.49	0.412	1.06	0.79–1.41	0.706	1.08	0.78–1.48	0.654	1.14	0.84–1.55	0.389	1.00	0.74–1.34	0.985	1.05	0.79–1.4	0.720	1.08	0.78–1.5	0.632
rs2853550	0.95	0.55–1.63	0.847	1.04	0.65–1.65	0.874	0.90	0.56–1.46	0.669	1.15	0.69–1.91	0.597	0.85	0.5–1.44	0.540	1.14	0.72–1.83	0.573	0.90	0.56–1.45	0.664	1.16	0.69–1.95	0.569
rs1143643	1.02	0.75–1.39	0.898	0.84	0.64–1.1	0.213	0.86	0.66–1.13	0.282	0.98	0.72–1.34	0.922	0.85	0.63–1.14	0.268	0.97	0.73–1.29	0.840	0.89	0.68–1.17	0.394	0.94	0.69–1.29	0.717
rs3136558	0.91	0.66–1.26	0.577	0.95	0.72–1.26	0.741	1.00	0.76–1.32	0.993	0.85	0.62–1.17	0.327	1.00	0.74–1.35	0.993	0.88	0.66–1.18	0.399	0.95	0.72–1.26	0.733	0.91	0.66–1.26	0.582
rs1143630	0.92	0.61–1.39	0.697	0.91	0.64–1.31	0.627	0.76	0.52–1.11	0.159	1.15	0.78–1.7	0.476	0.72	0.47–1.09	0.117	1.11	0.77–1.59	0.568	0.89	0.62–1.27	0.515	0.96	0.64–1.46	0.863
rs1143627	1.04	0.77–1.42	0.783	1.11	0.85–1.46	0.433	1.38	1.05–1.81	0.023	0.78	0.57–1.06	0.113	1.41	1.05–1.89	0.023	0.86	0.65–1.14	0.292	1.14	0.87–1.49	0.341	1.01	0.74–1.38	0.968
rs16944	0.93	0.68–1.27	0.639	0.89	0.68–1.17	0.407	0.73	0.56–0.96	0.026	1.22	0.9–1.66	0.202	0.71	0.53–0.96	0.025	1.12	0.85–1.49	0.424	0.85	0.65–1.11	0.239	1.00	0.73–1.37	1.000
rs1143623	0.95	0.69–1.31	0.759	0.97	0.741.28	0.821	0.80	0.6–1.06	0.112	1.24	0.91–1.7	0.166	0.86	0.63–1.15	0.305	1.07	0.8–1.42	0.649	0.91	0.7–1.2	0.522	1.04	0.76–1.43	0.813

SNP: single nucleotide polymorphism, OR: odds ratio, CI: confidence interval, HWE: Hardy–Weinberg equilibrium , *p* ≤ 0.05 indicates statistical significance.

**Figure 1:**
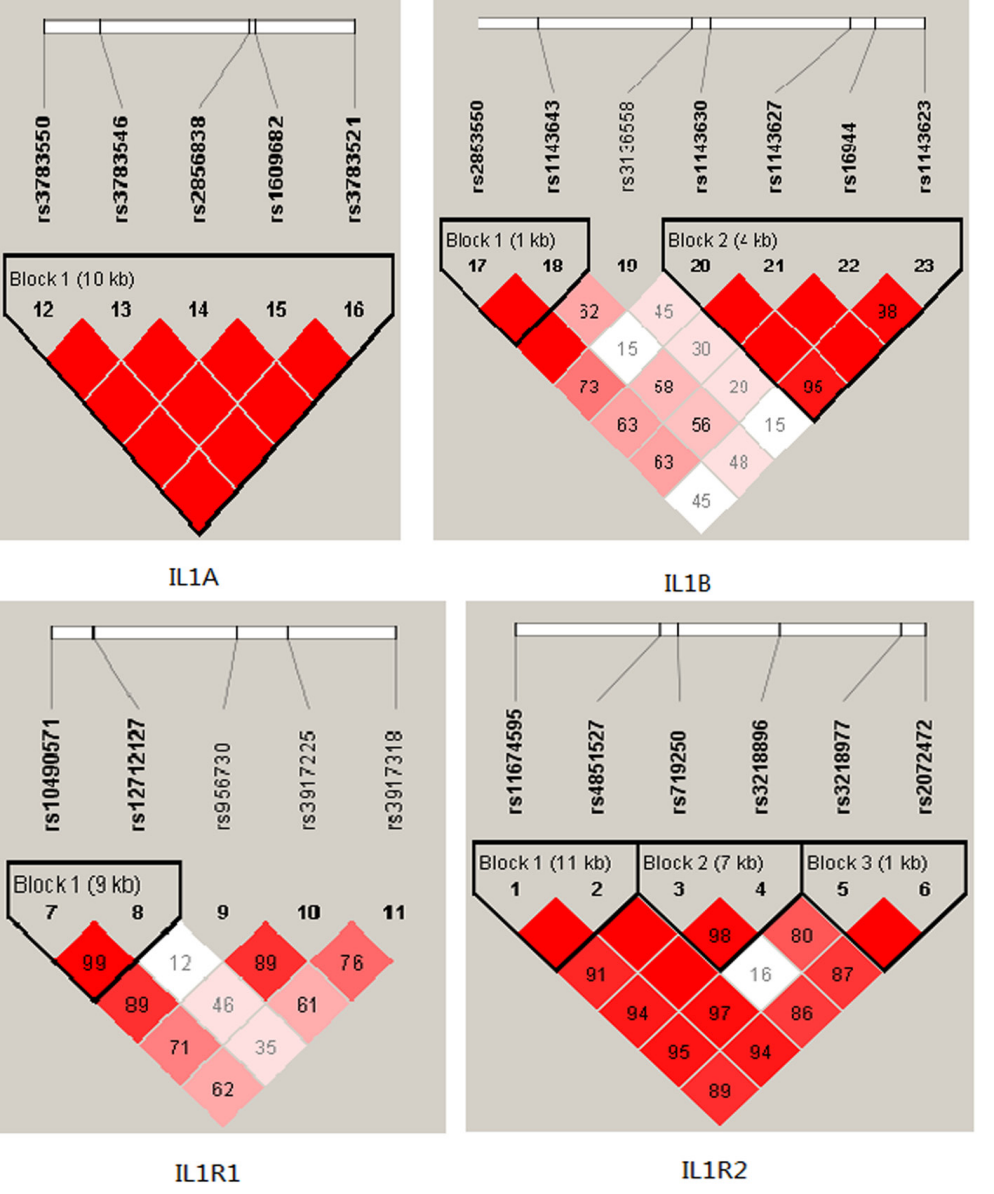
D’ linkage map for the SNPs in *IL1A,IL1B, IL1R1, IL1R2.*

Among controls, this SNP was found to be in high LD in IL1A and IL1B (all pairwise *r*^2^ > 0.80) in Figure 1. The association between the IL1A and IL1B haplotype and the risk of BC were analysised, however, we did not find any statistical evidence for the risk of breast cancer in our study.

## DISCUSSION

In this study, we focused on a possible inflammation role, we investigated the association of twenty-two polymorphisms in IL1A, IL1B, IL1R1 and IL1R2 gene with BC in 530 patient compared to628 normal women as the control group. Our results suggested that rs10490571 in IL1R1 and rs16944, rs1143623 in IL1B were associated with BC risk in Chinese Han population.

Cytokines are small molecules secreted by cells in response to specific stimuli and change the behavior of the same or other cells. Multifunctional cytokines are closely related to the development of inflammatory and immunological responses which play a key role in the pathogenesis of autoimmune and malignant diseases, making them important candidate genes in BC [20, 21]. The single nucleotide polymorphisms in a particular candidate gene are thought to affect the expression and / or activity of the encoded protein, thereby inducing solid cancer, particularly breast cancer [22]. The polymorphism of the cytokine gene regulatory region may alter its expression level associated with the immune response [23, 24]. IL-1 family of cytokines is encoded by two separate genes, IL1A and IL1B are located within a 430 kb region on chromosome 2q14.2 in a cluster and also contains the genes for IL1 receptors type 1 and 2 (IL1R1 and IL1R2), which are known as t the key regulation of inflammation and immune response to multifunctional cytokines and thought that almost affects all types of cells [15]. IL-1 has been recently suggested to play a role in the development of breast cancer. IL-1A and IL-1B are produced by monocytes, macrophages and epithelial cells and are involved in various processes such as modifying host response to microbial invasion, tissue injury and inflammation [25]. Both IL-1A and IL-1B cause inflammation, but more importantly, they induce the expression of proinflammatory genes, such as cyclooxygenase type 2, inducible nitric oxide synthase, and other cytokines/chemokines [26]. There is accumulating evidence indicating the presence of a peritumoural inflammatory infiltrate in BC, which may reflect, at least in part, an antitumour immune response.IL-1A and IL-1B expression was increased in human breast cancer tissues. Present studies mostly focused on the associations between IL1B gene polymorphisms and BC risk, for rs1143627, in our study we found the polymorphisms was associated with BC risk before adjusted for age, however, by adjusted for age we did not found significant. Ito *et al*. [27] first reported rs1143627 is significantly associated with breast cancer risk (CC vs.TT: OR = 1.82, 95% CI = 1.03–3.23) in the Japanese. Another case–control study by Liu *et al*. [28] supported the association in a Chinese population (CC vs. TT: adjusted OR = 1.72, 95% CI = 1.16–2.54). Hefler *et al*. [29] reported the rs16944 were associated with BC in European. Meanwhile, in our study also found the same result, individuals carrying “A” alleles to reduce the risk of breast cancer. However, up to now, there were fewer reports on the association between rs10490571 in IL1R1 polymorphisms and BC risk. Previous studies have reported with IGA nephropathy, osteoarthritis has significant correlation, in our study we found significant correlation could be found between rs10490571 polymorphism and BC risk. However, the results remained controversial partially because of small sample size, the difference in the genotype distribution by ethnicity, study design, assay characteristics and so on.

In conclusion, we showed a correlation of polymorphic markers within the proinflammatory-cytokine IL-1 gene locus with the risk in developing breast cancer.

Taken together with our finding that IL1B, IL1R1 gene three SNP are also associated with the risk for the disease, we suggest that inflammation via innate and adaptive immunity contributes to multifactorial hereditary predisposition to pathogenesis of the breast cancer.

## MATERIALS AND METHODS

### Subjects

A total of 530 BC patients and 628 healthy controls were consecutively recruited between June 2012 and July 2016 at the First Affiliated Hospital of Xi’an Jiaotong University, People's Republic of China. The controls had no family history of BC and all had been clinically confirmed and/or had a recent mammogram confirming that there was no detectable BC at the time of sampling. Clinicopathological parameters including age, histological subtype, TNM stage, tumor grade, lymph node metastasis, age at menarche, menopause status, number of pregnancies, number of deliveries, and family history of cancer were evaluated. Those who signed an informed consent form were asked to complete a self-administered questionnaire and to provide a 5 ml peripheral blood sample.

In the case-control design, we selected 23 SNPs in the IL-1A, ILB, IL1R1, IL1R2 polymorphisms in BC patients from China. These SNPs from DbSNP database (http://www.hapmap.org/index.html.en) and SNP Consortium database for analysis and each had minor allele frequency (MAF) of >5% in Chinese Han population. DNA was isolated from Whole blood were used the GoldMag-Mini Whole Blood Genomic DNA Purification Kit (GoldMag Co. Ltd. Xi’an City, China) extracted. Genotypes for SNPs were determined by Sequenom MassARRAY .We used a NanoDrop 2000 (Gene Company Limited) were measured DNA concentrations. We used Sequenom MassARRAY Assay Design 3.0 Software to design a Multiplexed SNP MassEXTEND assay [30]. The PCR primers for each SNP are shown in Table 2. Data management and analysis was performed using the Sequenom Typer 4.0 Software [30, 31].

### Differences in demographic characteristics, selected variables and genotype

Distribution, allele frequencies were analysed using the chi-square test or *t*-test. The associations between IL1 polymorphism and breast cancer risk were estimated using logistic regression to compute odds ratios (OR) and 95% confidence intervals (CI). computing the odds ratios (ORs) and their 95% confidence intervals (CIs) from both univariate and multivariate logistic regression analyses. Hardy–Weinberg equilibrium was tested by a goodness-of-fit v2 test to compare the observed genotype frequencies to the expected frequencies among the control subjects. Two sided *P* value of less than 0.05 was considered to be significant.
